# Magnitude and associated factors of substance use among pregnant women attending antenatal care in public hospitals of eastern Ethiopia

**DOI:** 10.1186/s12888-021-03078-5

**Published:** 2021-02-15

**Authors:** Metsihet Tariku Fetene, Kedir Teji, Nega Assefa, Wubet Alebachew Bayih, Genet Tsehaye, Habtamu Shimels Hailemeskel

**Affiliations:** 1 School of Nursing and Midwifery, Jigjiga University, Jigjiga, Ethiopia; 2 School of Nursing and Midwifery, Haramaya University, Harar, Ethiopia; 3 College of Health Sciences, Debre Tabor University, Debre Tabor, Ethiopia

**Keywords:** Substance use, Magnitude, Pregnant women

## Abstract

**Background:**

Use of substances like alcohol, tobacco and khat during pregnancy can bring miscarriage, prematurity, neurodevelopmental problems, sudden infant death syndrome and others. There are limited studies on the magnitude and associated factors of substance use among pregnant women in Eastern Ethiopia. Therefore, the aim of this study was to assess the magnitude and associated factors of substance use among pregnant women attending antenatal care in public hospitals of Easttern Ethiopia, 2019.

**Method:**

Hospital based cross-sectional study was employed on 510 pregnant women attending ANC at public hospitals of Eastern Ethiopia (Jigjiga, Dire Dawa and Harar towns). Data were collected from the study participants that were selected using systematic sampling method from each public hospital. The data were collected through interviewer administered structured questionnaire. Binary logistic regressions with 95% confidence interval were used to determine the degree of association between covariates and outcome variable. Multicollinearity between independent variables by using the standard error was checked. The goodness of fit was tested by Hosmer-Lemeshow statistic and Omnibus tests.

**Results:**

Out of 526 participants, a total of 510 study participants were involved in this study thereby making a response rate of 96.9%. In this study, the magnitude of substance use among pregnant women attending ANC was 26.5% (95% CI: 22.7, 30.6%). Among the overall pregnant mothers, 100 (19.6%) chewed khat, 48 (9.4%) drank alcohol, 12 (2.4%) used tobacco products and 28(20.7%) were dual substance users. Pre pregnancy substance use (AOR = 27.25, CI: 14.107–52.66), partner substance use (AOR = 3.704 CI: 1.839–7.464), family substance use (AOR = 3.447 CI: 1.69–7.031) and the amount of monthly household income (AOR = 3.397, 95% CI: 1.316–8.766) were found to be statistically significant and positively associated with substance use during pregnancy.

**Conclusion:**

The magnitude of antenatal substance use in the study area was 26.5%. Pre- pregnancy substance use, partner substance use, monthly house hold income and family substance use were found to be positively associated with substance use during pregnancy. Therefore, health education which is inclusive of child bearing age women with their partner and family may be helpful to decrease antenatal substance use during pregnancy.

**Supplementary Information:**

The online version contains supplementary material available at 10.1186/s12888-021-03078-5.

## Background

The term “substance use” refers to the use of any substance which changes the way the body functions, mentally, physically or emotionally and it includes substances such as alcohol, tobacco, caffeine, illegal drugs, prescription drugs, inhalants and solvents [1]. The type of substance being used and the degree of use, as well as the point of exposure, all influence the effects of drug use in pregnancy. Globally, the most frequently used substance in pregnancy is tobacco followed by alcohol, cannabis and other illicit substances [2].

Tobacco is one of the common types of substances used by pregnant women. Tobacco use among pregnant women was reported to be 22% in Nepal [3], 37.1% in Brazil [4], 36.8% in South Africa [5] and 8.2% in Burundi [6]. In Ethiopia, a study conducted in Jimma [7] revealed that the prevalence of substance use among pregnant women was 37.9, and 2.7% of whom were active tobacco smokers. Furthermore, another study in Butajira, South Central Ethiopia [8] showed 60.1% prevalence of substance use during pregnancy; and 23.2% of these substance users were passive tobacco smokers, but none were active tobacco smokers.

Alcohol is the other common substance used by pregnant women. The Prevalence of alcohol drinking among pregnant women was 8.5% in the United States [9], 10.0% in Canada [10], 5.5% in Sweden [11], 23% in Brazil [12] and 0.9% in Japan [13]. Furthermore, among Sub-Saharan countries, the prevalence was reported to be 20.2% in South Africa [5], 22.6% in South-Eastern Nigeria [14], 48% in Ghana [15] and 29.5% in Uganda [16]. In Ethiopia, studies about the prevalence of alcohol drinking among pregnant women at different towns of the country showed 29.7% in Jimma [7], 34% in Bahir Dar [17], 37.1% in Addis Ababa [18] and 10.0% in Butajira [8].

Scientifically, *khat* is known as *Catha edulis*, a name which indicates that the leaves of the plant are edible. The plant is indigenous to Ethiopia. Cathinone is the most important chemical found in *khat*, which is believed to have the same effect as amphetamine, a stimulant of the central nervous system [19]. *Khat* chewing among *khat* cultivating countries was high. For example, a recent Ethiopian study [20] about the correlates of khat use during pregnancy showed 19.1% prevalence of khat use in the country. Furthermore, the prevalence of khat chewing during pregnancy at different Ethiopian towns was reported to be 65.8% in Jimma [7], 9.9% in Gedeo zone [21] and 35.8% in Butajira [8]. According to CSA and ICF, khat chewing was more common in Harari and Dire Dawa regions of Ethiopia, where Khat is located [17]. Furthermore, a systematic review and metaanalysis of studies about Khat chewing during pregnancy revealed that the pooled magnitude of antenatal khat chewing was 20% in Ethiopia [22].

The use of alcohol, tobacco and khat during pregnancy remains a significant public health problem because their use can lead to several harmful maternal and neonatal outcomes [23]. Prenatal use of these substances can bring miscarriage, stillbirth, preterm birth, low birth weight, neurodevelopment problems including fetal alcohol spectrum disorders (FASDs), sudden infant death syndrome (SIDS), colic with uncontrollable crying, asthma and obesity in childhood. Moreover, injuries/violence, unintended pregnancy, sexually transmitted diseases, fertility problems, heart disease and cancer can be some of the adverse maternal consequences resulted from substance use [13, 24–26].

Alcohol is a commonly used substance during pregnancy despite its teratogenic potential. It can disturb fetal development, especially affecting the fetal central nervous system with potentially severe lifelong consequences [23]. No amount of alcohol consumption can be considered safe during pregnancy. Damage can occur in the earliest weeks of pregnancy, even before a woman knows that she is pregnant. The damage from prenatal alcohol exposure can be of birth defects and neurodevelopmental abnormalities which can be prevented [27].

The effect of *khat* chewing during pregnancy increases with its increased frequency and duration of use [20, 28]. Khat chewing during pregnancy is associated with Anemia [29], stillbirths and impaired lactation [30]. A study at primary care centers in Ethiopia showed that current and former khat users had higher levels of depressive symptoms and distress [20]. Other case-control studies in Ethiopia also showed that maternal history of khat chewing was associated with low birth weight [31, 32].

When a woman smokes cigarettes during pregnancy, her fetus is exposed to many harmful chemicals. Nicotine is only one of 4000 toxic chemicals that can pass from a pregnant woman to her fetus. Nicotine causes blood vessels to narrow, so less oxygen and fewer nutrients reach the fetus [23]. Smoke of the cigarette has both maternal and neonatal adverse effects. The adverse maternal effects include maternal depression during pregnancy [33] while the fetal effects include an increased risk of strabismus in the offspring [34], clubfoot [35], low birth weight and preterm births [36]. Furthermore, during adolescence and adulthood, antisocial behaviors [37], increased risk of wheezing in children [38] and risk of congenital heart defects can be ensued [39].

Different factors were found to increase the likelihood of substance use in pregnancy. Maternal age, nulliparity, educational status, occupation, history of pre-pregnancy substance use, and lack of awareness about the harmful fetal effects of substance use have been found to be significantly associated with substance use during pregnancy [7, 14]. Furthermore, socialization is among contributing factors to increase the likelihood of substance use. This is because a socialized woman tends to feel social inclusion and more freedom [40–44] thereby enhancing the desire to interact with one another which is among the core reasons to engage in substance use.

Consequences of substance use are preventable through different strategies like CDC screening and counseling strategies for alcohol drinking and strengthening WHO Framework Convention on Tobacco Control (FCTC) for users. CDC gives stress for health care providers to help women avoid drinking too much, and avoid alcohol during pregnancy [25, 45]. Additionally, there are pharmacological and behavioral interventions in the treatment of substance use disorder. But, only a small number of therapies are effective for substance use in pregnancy, which primarily involves behavioral counseling [46]. As with alcohol, behavioral counseling which consists of Contingency management (CM), Motivational interviewing (MI) and Cognitive Behavioral Therapy (CBT) is found to be effective. Contingency management has been shown to reduce both prenatal alcohol use and smoking [47, 48]. Ethiopia is among countries that implement the WHO MPOWER policy package which is incorporated in FCTC [38]. However, the implementation has been challenged by shortage of mental health specialists and poor quality of services on substance use disorders in the past years [49]. Therefore, this study will give an insight in identifying the possible enabling factors of antenatal substance use where actions can be set.

Despite the teratogenic impacts, alcohol consumption, khat chewing and cigarette smoking are practiced in Ethiopia [50]. Moreover, khat is highly cultivated and marketed in eastern Ethiopia. However, to the authors’ best searching effort and knowledge, there is limited prior data on the prevalence and associated factors of substance use among pregnant women attending antenatal care at public hospitals of eastern Ethiopia. Therefore, the aim of this study was to assess the prevalence and associated factors of substance use among pregnant women attending antenatal care at public hospitals in eastern Ethiopia. Findings of this study can help clinicians, policy planners and decision makers by showing the burden and determinants of substance use during pregnancy. Furthermore, the findings will be used as baseline information for further studies.

## Methods

### Study area and study period

This study was conducted among pregnant women attending ANC at public hospitals found in Jigjiga, Harar and Dire Dawa towns of Eastern Ethiopia, from February to March 2019. Jigjiga town is the capital city of Somali Regional State, located 635 km from Addis Ababa, the capital of Ethiopia. The town has one referral hospital and one general hospital. Harari regional state is located in the Eastern Ethiopia at a distance of 526 km away from Addis Ababa. There are 2 public hospitals in Harari region. Dire Dawa is among the Ethiopian Federal administrative towns located in the Eastern part of the country at a distance of 515 KMs from Addis Ababa. There are 2 public hospitals in Dire Dawa.

### Study design and participants’ characteristics

An institution based cross- sectional study was done on pregnant women attending ANC at public hospitals located in Jigjiga, Dire Dawa and Harar towns of Eastern Ethiopia.

### Sample size determination and sampling procedure

Using double population proportion formula, the desired sample of 526 pregnant women was calculated from Open EPI INFO version 7 software by considering 95% level of confidence, power of the study 80%, exposed to unexposed ratio of 1:1, out come in the unexposed group 36.7% and AOR of 1.71 as of a study conducted in Bahir Dar, Northern Ethiopia [17].

**Fig. 1:**
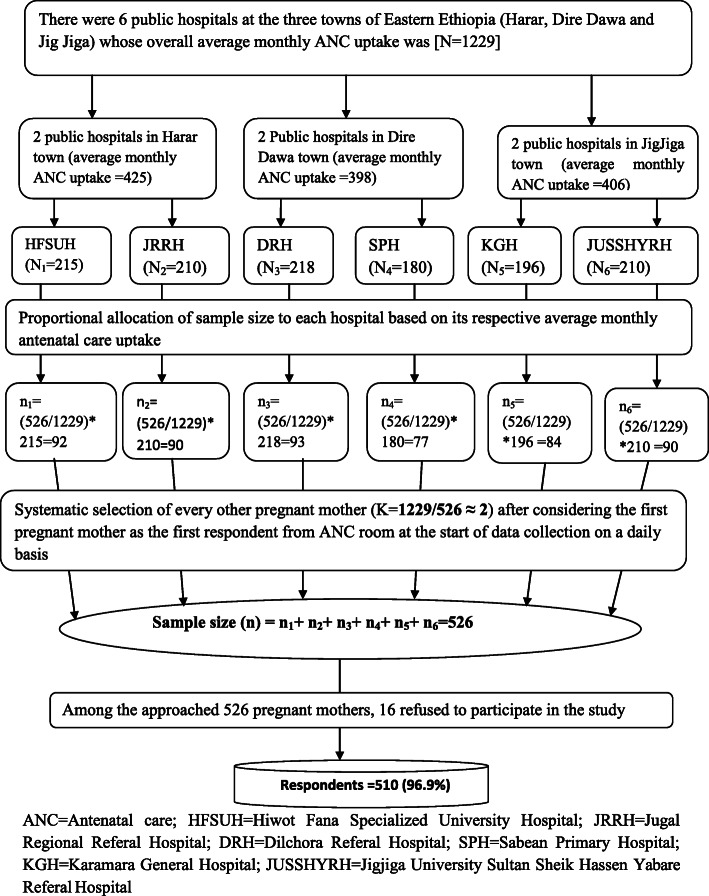
A flow diagram that illustrates sampling procedure of pregnant mothers attending antenatal care at public hospitals of Dire Dawa, Harar and Jig Jiga towns, Eastern Ethiopia, 2019

The study involved all the six public Hospitals found in three towns of the study area. The average monthly antenatal care attendants for each town and hospital was: 425 from Harar town public Hospitals (i.e. 215 from Hiwot Fana Specialized University Hospital and 210 from Jugol Regional Referral Hospital), 398 from Dire Dawa town public Hospitals (i.e. 218 from Dilchora Referral Hospital and 180 from Sabiyan Primary Hospital) and 406 were from Jigjiga town public Hospitals (i.e. 196 from Karamara General Hospital and 210 from Jigjiga University Sultan Sheik Hassen Yabare Referal Hospital). The sum of average monthly Ante Natal Care uptake (ANC) of the six public hospitals in the three towns was considered as source population (N=N_1_ + N_2_ + N_3_ + N_4_ + N_5_ + N_6_ = 1229). Then, the calculated sample size was allocated for each public hospital proportionally based on its respective average monthly ANC uptake. As a result, 90 pregnant mothers were distributed for Jigjiga University Sultan Sheik Hassen Yabare Referal Hospital (JUSSHYRH), 84 for Karamara General Hospital (KGH), 92 for Hiwot Fana Specialized University Hospital (HFSUH), 90 for Jugol Regional Referral Hospital (JRRH), 77 for Sabiyan Primary Hospital (SPH) and 93 for Dilchora Referral Hospital (DRH). The pregnant woman who was getting care inside the ANC room while the data collectors arrived at the room was considered as the first to be interviewed. Then, systematic sampling technique was employed to select every other (K = 1229/526 = 2) eligible pregnant mother from each public hospital in the three towns (Fig. 1). Among the 526 mothers approached, there were 16 non-respondents (4 mothers were from Dire Dawa town, 5 from Jig Jiga town and 7 from Harar town).

### Measurement and data collection procedure

The data collection tool was a structured questionnaire consisting of socio-demographic characteristics, obstetrics related characteristics, substance use related characteristics (substance use before and during current pregnancy), personal and social factors. The questionnaire was first prepared in English and then translated to Afan Oromo, af- somali and Amharic languages to facilitate understanding and ensure consistency during administration (Additional file 1). Data were collected by 12 diploma midwives under the supervision of five BSc midwives. An exit interview was made to every other pregnant mother after getting antenatal care in each hospital. The interviews were conducted on working days on a daily basis during the study period. To assure data quality, 2 days of training (1 day theoretical and 1 day practical) was given for data collectors and supervisors. Pretest was done on 26 pregnant mothers (5% of the total sample size) attending ANC at Gursum health center to assess simplicity, flow and consistency of the questionnaire. Some modifications were made based on the pretest findings. Close supervision was done by the supervisors and the principal investigator to ensure for data completeness and consistency.

### Operational definition

Substance use during current pregnancy was defined as self-report of exposure to at least one of the three substances (alcohol or khat or tobacco) during current pregnancy prior to the interview irrespective of its dose and frequency (yes/no) [51, 52].

Alcohol use during current pregnancy was defined as ever use of drinks with industrially prepared alcoholic content (ethanol or ethyl content) or locally prepared ones, like *Teji, Areki and Tela* during current pregnancy irrespective of its dose and frequency (yes/no) [17, 23].

Tobacco use during current pregnancy was defined as ever use of any of the tobacco products during current pregnancy. The use of cigarette or other forms of smoking tobacco like *shisha/hookah* and *bidi* and non-smoking like snuff or chewing tobacco during current pregnancy irrespective of its dose and frequency (yes/no) [42].

Khat use during current pregnancy was defined as ever chewing of khat during current pregnancy irrespective of its dose and frequency (yes/no) [20, 22].

Pre-pregnancy substance use was defined as self-report of the use of at least one of the three substances of any dose and frequency (alcohol, khat or tobacco product of any type) prior to current pregnancy in the last 12 months (yes/no) [52].

Positive to intimate partner violence was considered if there was even one “yes” response among the series of questions.

A few times represent the 2–5 times frequency of intimate partner violence [53].

Many times represent more than 5 times frequency of intimate partner violence [53].

Intimate partner refers to husband, ex-husband, or boyfriend [54].

### Statistical analysis

The dependent variable of the study was substance use during pregnancy (yes/no) whereas the independent variables were maternal age, residence, educational status, marital status, average monthly household income, occupation, pre-pregnancy substance use, maternal awareness about the adverse effects of substance use during pregnancy, family history of substance use, intimate partner violence and obstetrics related factors including gestational age, pregnancy plan, gravidity, parity and ANC follow up. The collected data on the aforementioned variables were coded, cleaned, edited and double entered into epidata version 3.1 after which it was exported to SPSS version 21 software for further analysis. Considering the exported data on the variables, the assumptions for binary logistic regression model were first checked. Then, bivariable logistic regressions were carried out to identify as many candidate independent variables as possible (*p* < 0.25) for multivariable analysis. Following bivariable analysis, multivariable logistic regressions were performed using those candidate variables to investigate statistically significant predictors of substance use during pregnancy. Finally, variables whose *p* value less than 0.05 (*p* < 0.05) from multivariable logistic regressions were declared as statistically significant using adjusted odds ratio of 95% CI. Multi-collinearity between the study variables was diagnosed using standard error and correlation matrix. Hoshmer-Lemeshow statistic and Omnibus tests were also performed to test for model fitness.

### Ethical consideration

Before the actual data collection, ethical clearance was obtained from Institutional Health Research Ethics Review Committee of the College of Health and Medical Science in Haramaya University. Then, permission letter was obtained from the College of Health and Medical Sciences of the university and provided to Dire Dawa city administration, Jigjiga and Harari regional health bureau. Informed voluntary verbal consent was obtained from all study participants prior to the interview. All the participants were above 16 years old and hence parental consent wasn’t required. The study participants’ confidentiality, privacy, and the right to withdraw from the study at any time were ensured.

## Results

### Socio-demographic characteristics

Out of 526 pregnant women approached during the study period, a total of 510 study participants were involved in this study thereby making a response rate of 96.9%. The mean age of study participants was 26.63(±5.12 SD) years and majority of the participants, 340(66.7%) were muslim by religion and 430 (84.3%) were urban residents. Almost all, 464(91%) were married and nearly one third of the respondents, 161 (31.6%) didn’t attend formal education. Above half of the respondents, 274(53.7%) were housewives (Table 1).

**Table 1:** Socio-demographic characteristics of pregnant women attending ANC in public hospitals of Eastern Ethiopia, 2019(*n* = 510)

Variables	Frequency	Percentage
Town		
Dire Dawa	166	32.6
Harar	175	34.3
Jig Jiga	169	33.1
Age		
16–24	181	35.5
25–29	167	32.7
> 29	162	31.8
Religion		
Muslim	340	66.7
Orthodox	142	27.8
Others^a^	28	5.5
Residence		
Urban	430	84.3
Rural	80	15.7
Ethnicity		
Oromo	185	36.3
Somali	173	33.9
Amhara	91	17.8
Others^b^	61	11.9
Marital status		
Married	464	91
Other^c^	46	9
Level of education		
No formal education	161	31.6
Elementary	60	11.8
Secondary school	160	31.4
10+	129	25.3
Occupation		
Unemployed	378	74.1
Student	47	9.2
Employee	85	16.7
Monthly household income		
< 1500	84	16.5
1500–3499	209	41
3500–4999	90	17.6
≥5000	127	24.9

Others^a^:Protestant, Catholic, Others^b^:Gurage, Tigre, Hadiya, Others^c^: Widowed, Single, Divorced, Cohabitated,

### Obstetrics and health related characteristics

The median gestational age of pregnant women during the current pregnancy was 25 weeks and 201(39.4%) of whom were in the second trimester. Regarding gravidity, the median number of pregnancy was 3 with a minimum of one pregnancy and a maximum of 11 pregnancies. Almost three-fourth of the participants, 374 (73.3%) were multigravidous. The median parity of the participants was 2 with a maximum of having 10 children. The current pregnancy status for more than three-fourth 420(82.4%) of the mothers was planned. Two third of all pregnant women 338 (66.3%) had more than one ANC visits and the median number of ANC visit was 2 (Table 2).

**Table 2:** Description of obstetric-related characteristics among pregnant women attending ANC in public hospitals of Eastern Ethiopia, 2019(n = 510)

Variables	Category	frequency	Percentage
Gestational age			
	1st trimester	114	22.4
	2nd trimester	201	39.4
	3rd trimester	195	38.2
Gravidity			
	Primigravida	136	26.7
	Multigravida	374	73.3
Number of children			
	≤ 2 children	357	70
	> 2 children	153	30
Pregnancy plan			
	Planned	420	82.4
	Unplanned	90	17.6
ANC visits			
	1 visit	172	33.7
	> 1 visits	338	66.3

### Personal and social related characteristics

One hundred ninety six pregnant mothers (38.4%) used substance/s before their current pregnancy, and 141(71.9%) of whom chewed khat. Furthermore, the use of Khat was reported among pregnant women’s partner 313(61.4%) and family 64 (32.75%). During pre-pregnancy period, 160(82.1%) of the pregnant mothers used only one type of substance. Around one third 150 (29.3%) of the respondents experienced violence by their intimate partners (Table 3).

**Table 3:** Behavioral and social characteristics of pregnant women attending ANC in public hospitals of Eastern Ethiopia, 2019

Variables	Frequency	Percentage
Pre-pregnancy SU(n = 510)	196	38.4
Type of pre pregnancy substance. Used		
Khat	141	71.9
Alcohol	64	32.7
Tobacco	26	12.8
Number of substance type (*n* = 196)		
1	160	82.1
≥ 2	35	17.1
Partner SU(n = 510)	313	61.4
Substance type used by partner		
Khat	257	82.1
Alcohol	73	23.3
Tobacco	70	21.5
Family SU(n = 510)	328	64.3
Substance type used by family		
Khat	276	79.8
Alcohol	67	19.4
Tobacco	41	11.8
IPV(n = 510)		
Yes	150	29.3
No	360	70.7
Awareness on harmful effects of SU		
Aware	410	74.9
Not aware	100	25.1

*SU* substance use

### Self-reported substance use status during the current pregnancy

Out of 510 pregnant women attending antenatal care clinics, about one fourth [135 (26.5%) (95% CI: 22.7, 30.6%)] used substance/s during their current pregnancy. From the reported substances, Khat 100 (19.6%) was the most repeatedly used type of substance followed by alcohol 48(9.4%) and tobacco 12(2.4%).

Cross tabulation of substance use during pregnancy versus maternal awareness of the teratogenic effects of substance use revealed that one quarter of the substance users (126(24.7%) didn’t have the awareness about the teratogenic effects of antenatal substance (Table 4).

**Table 4:** Cross tabulation of maternal awareness of the teratogenic effects of substance use during pregnancy versus substance use during pregnancy

		Substance use during pregnancy		Total (%)
		User (%)	Not user (%)	
Maternal awareness about the teratogenic effects of substance use during pregnancy	Not aware	126(24.7%)	2(0.4%)	100(25.1%)
	Aware	9(1.8%)	373(73.1%)	410(74.9%)
Total		135(26.5%)	375(73.5%)	510(100.0%)

### Factors associated with substance use during pregnancy

Findings of this study revealed that pregnant women with monthly household income between 3500 and 4999 Ethiopian birr were three times (AOR = 3.40, 95% CI: 1.32–7.76) more likely to use a substance during the current pregnancy. Additionally, pregnant women with pre-pregnancy substance use history had 27 times (AOR = 27.26, CI: 14.10–52.66) higher odds of association with substance use during their current pregnancy as compared to their counter parts. Furthermore, pregnant women whose partners used substances had four times (AOR = 3.70 CI: 1.84–7.46) higher odds of association with substance use during current pregnancy. The odds of substance use among pregnant mothers whose families used substances were 3.4 times (AOR = 3.45 CI: 1.69–7.03) higher than those whose family didn’t use any substance. However, age, marital status, educational status and pregnancy plan status were not found to be significantly associated (Table 5).

**Table 5:** Factors associated with substance use during current pregnancy among pregnant women attending ANC in public hospitals of Eastern Ethiopia, 2019(n = 510)

Variables	Substance use		COR(95% CI)	AOR(95% CI)
	Yes (%)	No (%)		
Age				
15–24	35(19.3)	146(80.7)	0.49(.30–0.81)	1.23(0.54–2.78)
25–29	47(28.1)	120(71.9)	0.81(0.50–1.29)	0.80(0.39–1.65)
> 29	53(32.7)	109(67.3)	1	1
Residence				
Urban	109(25.3)	321(74.7)	1	1
Rural	26(32.5)	54(67.5)	1.42(0.85–2.38)	0.88(0.38–2.02)
Marital status				
Married	122(25.4)	358(74.6)	0.45 (0.21–0.94)	0.89(0.25–3.11)
Single	13(43.3)	17(56.7)	1	1
Educational status				
No formal education	45(28)	146(72)	1.7 (0.97–2.98)	1(0.35–2.86)
Elementary	20(33.3)	40(66.7)	2.19 (1.09–4.39)	0.80(0.28–2.28)
Secondary school	46(28.8)	114(71.2)	1.77 (1.00–3.09)	1.27(0.50–3.22)
10+	24(18.6)	105(81.4)	1	1
Occupation				
Unemployed	104(27.5)	274(72.5)	0.96 (0.57–1.63)	0.78(0.33–1.84)
Student	7(14.9)	40(85.1)	0.44 (0.17–1.13)	0.48(0.10–2.19)
Employee	24(28.2)	61(71.8)	1	
Monthly household income				
< 1500	25(29.8)	59(70.2)	1.92 (1.00–3.67)	1.254(0.46–3.42)
1500–3499	60(28.7)	149(71.3)	1.82 (1.06–3.13)	1.921(0.83–4.42)
3500–4999	27(30)	63(70)	1.94 (1.02–3.67)	3.4(1.32–8.77)*
≥ 5000	23(18.1)	104(81.9)	1	1
Gravidity				
Primigravida	29(21.3)	107(78.7)	1	1
Multigravida	106(28.3)	268(71.7)	1.46 (0.91–2.33)	1.87(0.86–4.06)
No of children				
0–2	87(24.4)	270(75.6)	0.70 (0.46–1.07)	0.63 (0.31–1.30)
> 2	48(31.4)	105(68.6)	1	1
Plan status				
Planned	101(24)	319(76)	1	1
Not planned	34(37.8)	56(62.2)	1.92(1.18–3.10)	1.60(0.75–3.43)
No of ANC				
= 1	40(23.3)	132(76.7)	0.77 (0.51–1.19)	0.90(0.43–1.87)
> 1	95(28.1)	243(71.9)	1	1
Pre-pregnancy use				
Yes	118(60.2)	78(39.8)	26.43(15.00–46.56)	27.25(14.11–52.66)***
No	17(5.4)	297(94.6)	1	1
Partner SU				
Yes	118(37.7)	195(62.3)	6.41(3.71–11.08)	3.70(1.84–7.46)***
No	17(8.6)	180(91.4)	1	1
Family SU				
Yes	118(36)	210(64)	5.45(3.15–9.43)	3.45(1.69–7.03)**
No	17(9.3)	165(90.7)	1	**1**
Intimate partner violence				
Yes	58(38.9)	91(61.1)	2.34 (1.55–3.55)	0.77(0.42–1.41)
No	77(21.4)	283(78.6)	1	1

*AOR* adjusted odds ratio, *COR* crude odds ratio: *Significant at *P* < 0.05, **Significant at *P* < 0.01, ***Significant at *P* < 0.001, 1 = Reference

## Discussion

In this study, the overall magnitude of substance use among pregnant women was 26.5%. From the overall sample of pregnant mothers, 19.6% chewed khat, 9.4% drank alcohol and 2.4% smoked cigarette during pregnancy. Furthermore, pre pregnancy substance use, partner substance use, family substance use and the amount of average monthly household income were factors of statistical significance having positive odds of association with substance use during pregnancy.

The overall magnitude of substance use among pregnant women in the study area 26.5% was lower than the findings of studies conducted in Jimma 37.9% [7] and Butajira 60.1% [8]. This may be due to the fact that our study included only khat, tobacco and alcohol use whereas that of the study in Butajira included caffeinated drinks in addition to khat, tobacco and alcohol use. Furthermore, difference in sample size and geographic location of the study subjects might have contributed roles for the variation.

In this study, the magnitude of khat chewing among pregnant mothers 19.6% accords with the study in Jimma 24.9% [7], but lower than the finding from Yemen 41% [55]. The discrepancy may be attributable to methodological differences between the studies. For example; the Yemeni study was a population based survey conducted at national level compared to our study which involved only a sample of mothers from Eastern Ethiopia. Moreover, the Yemeni study was conducted two decades ago.

The magnitude of alcohol drinking in the study area 9.4% was congruent with prior Ethiopian studies conducted in Jimma (11.3%) [7] and Butajira 10.0% [8]. However, it was lower than findings from South Africa 20.2% [5], South-Eastern Nigeria 22.6% [14], Uganda 29.5% [16] and Brazil 23% [12]. The variation may be due to socio-cultural differences between the study populations among the studies.

Regarding the magnitude of tobacco use, our finding 2.4% was consistent with prior Ethiopian studies at Jimma 1% [7] and Butajira 9.7% [8]. The consistence could be due to evidence [56] that witnesses the practice of cigarette smoking with khat chewing in Ethiopia. However, our finding was lower than studies conducted in Madagascar 15% [57], Nepal 22% [3], Brazil 37.1% [4] and South Africa 36.8% [5] which may be due to differences in study period, sample size and study population characteristics.

Pregnant mothers whose average monthly household income between 3500 and 4999 Ethiopia birr were three times more likely to use substance than those earning greater than 5000 birr. This may be due to the reason that pregnant women with lower income could use substance to cope with their increased stress and less access to alternative activities. Therefore, pregnant mothers with low household income should be empowered to engage in feasible income generating activities within their community. But, this finding was inconsistent with studies in Bahir Dar [17] and Jimma [7].

Pregnant mothers with history of pre-pregnancy substance use were 27 times more likely to use substance/s than those who did not use. This may be due to the fact that evidence witnesses the development of habit through repetition of a behavior [58] i.e. a mother who started substance use prior her pregnancy may develop a behavior to perpetuate their habit of using substance during pregnancy. Furthermore, lack of awareness about harmfulness of substance use during pregnancy might have played role for antenatal substance use. Thus, mothers who have pre-pregnancy substance use history should be given health education about the feto-neonatal and maternal adverse effects of substance use during their preconception care and ANC visits. This finding was consistent with studies in Nepal [3] and South-Eastern Nigeria [14].

Pregnant women whose partners used substance/s were four times more likely to use substance during their current pregnancy. This could be due to the fact that when a pregnant woman is repeatedly invited to use substance by her intimate partner, she becomes provoked to accept and consume the substance. As a result, pregnant women should be routinely screened to determine whether their partners use substance. Then, couple counseling should be given about maternal likelihood of using substance during pregnancy if her partner is substance user. The counseling should also involve about the catastrophic feto-neonatal effects (abortion, still birth, birth defects, birth asphyxia, prematurity, low birth weight etc) of substance use during pregnancy. This finding was consistent with studies in Bahir Dar [17] and Western South Africa [5].

Pregnant women whose families use substance were three times more likely to use substance during pregnancy than their counterparts. This may be due to the cultural asset in the study area where family plays great role in shaping the behavior of children especially on khat chewing [59]. This finding is consistent with a study in Jimma, Ethiopia [7], Nepal [3] and Brazil [4] . Hence, it is advisable to create community awareness about the short and long term effects of substance use during pregnancy to optimize pregnancy outcomes in the study setting and population.

Despite no statistical significance, greater number of substance users during pregnancy 126(24.7%) did not have the awareness about the teratogenic effects of their antenatal substance use; while among non-users only 2(0.4%) pregnant mothers were unaware. Therefore, antenatal health care providers in the study area should routinely provide health education to create maternal awareness about teratogenic effects of substance use during pregnancy.

### Strength and limitation

This study was multi-centered because it involved 6 hospitals from 3 different towns in eastern Ethiopia thus increasing representativeness of the sample. Furthermore, the study was based on primary data collected from direct maternal interview at antenatal care clinic thus generating more valid results than it would be if chart review was considered. Despite the aforementioned strengths, this study had some limitations. Firstly, the study was of cross-sectional design; hence, it didn’t show causal association between the independent and dependent variables. Moreover, maternal self-reported responses obtained during data collection were liable to social desirability biases resulting in underestimation of the magnitude of substance use. But, it was tried to minimize the bias by probing the respondents. Furthermore, there was difficulty in determining the dose of substance use.

## Conclusion

The magnitude of substance use in the study area was found to be a problem of public health importance. Moreover, pre-pregnancy substance use, partner substance use, low average monthly house hold income and family substance use were found to be significantly associated with higher odds of antenatal substance use. Therefore, health education programs about the feto-neonatal risks of antenatal substance use should be routinely given for women of child bearing age, pregnant women with their spouses, and family in general. Furthermore, the authors would like to recommend further research to identify the association between the dose of antenatal substance use and pregnancy outcomes.

## Supplementary Information

**Additional file 1.** Survey tool: A questionnaire containing Amharic, Afan Oromo, af- somali versions.

## Data Availability

Data will be available upon request from the corresponding author.

## Abbreviations

ACOG: the American College of Obstetricians and Gynacologists
ANC: Ante Natal Care
AODs: Alcohol and Other Drugs
AOR: Adjusted Odds Ratio
CDC: Centers for Disease Control and Prevention
CSA: Central Statistical Agency
DD: Dire Dawa
DHS: Demographic and Health Survey
EDHS: Ethiopia Demographic and Health Survey
FAS: Fetal Alcohol Syndrome
FASDs: Fetal Alcohol Spectrum Disorders
FCTC: Framework Convention on Tobacco Control
GATS: Global Adult Tobacco Survey
IPV: Intimate Partner Violence
LMICs: Low and Middle Income Countries
MPOWER: Monitor tobacco use and prevention policies, Protect people from tobacco smoke, Offer help to quite tobacco use, Warn about the dangers of tobacco, Enforce bans on tobacco advertising, promotion and sponsorship, Raise taxes on tobacco
NDPs: Neuro Developmental Problems
ROP: Recognition Of Pregnancy
SGA: Smalll for Gestational Age
SIDS: Sudden Infant Death Syndrome
SPH: Sabian Primary Hospital
SU: Substance Use
US: United States of America
WHO: World Health Organization
