# Supplementary material for: Magnitude and associated factors of substance use among pregnant women attending antenatal care in public hospitals of eastern Ethiopia
Source: BMC Psychiatry. 2021 Feb 15;21:96. doi: 10.1186/s12888-021-03078-5 (PMC7885430; doi:10.1186/s12888-021-03078-5)
Supplement: Supplementary file 1 — Additional file 1. Survey tool: A questionnaire containing Amharic, Afan Oromo, af- somali versions. [file 12888_2021_3078_MOESM1_ESM.docx]

**1. English version Questionnaire**

Date of interview (date/month/year):_____________________________

Code number of the questionnaire: _____________________________

Interviewer’s name & signature: Name: ___________________ Signature___________

Supervisor’s name & signature: Name: ___________________ Signature___________

| No. | **PartI. Socio demographic characteristics** | Response options | Skip |
| --- | --- | --- | --- |
| 101 | How old are you? | _____ years |  |
| 102 | What is your religion? | 1. Muslim  2. Orthodox  3. Protestant  4. Others------------ |  |
| 103 | Residence? | 1. Urban 2. Rural |  |
| 104 | Ethnicity? | --------------- |  |
| 105 | Current marital status? | 1. Married  2. Single  3.Widowed  4.Divorced  5.Cohabitated |  |
| 106 | Level of education? | 1.cann’t read and write  2.read and write  3.elementary school  4.secondary school  5.preparatory  6.College and above |  |
| 107 | What is your main occupation? | 1.Merchant  2.Housewife  3.Student  4.Employee  5.Others________ |  |
| 108 | Monthly household income | ___________ |  |
| **Part II. Obstetric related characteristics** | | |  |
| 201 | How many times ever be pregnant? | -------------- |  |
| 202 | How many children do you have? | -------------- |  |
| 203 | How many months pregnant are you now? | ------------- |  |
| 204 | Have you planned this pregnancy? | 1. Yes 2. No |  |
| 205 | How many ANC visits did you have? | ________ |  |

**Part III -Substance use related characteristics**

| **Awareness related questions** | | | |  | | | | |  | |
| --- | --- | --- | --- | --- | --- | --- | --- | --- | --- | --- |
| 301 | | Do you know the harmful effects of substance use during pregnancy? | | 1. Yes 2. No | | | | | 2→304 | |
| 302 | | If you say “yes” for question number “301” what harmful effects do you know? | | 1. spontaneous abortion yes no 2. low birth weight yes no 3. congenital heart diseases yes no 4. preterm labour yes no 5. neurodevelopmental problems yes no 6. other/specify | | | | |  | |
| 303 | | From where did you get this information? | | 1.health professionals 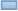yes 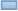 no  2. mass media 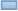 yes 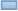 no  3.other person in the village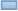 yes 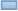no  4.I don’t remember | | | | |  | |
| **Pre pregnancy substance use** | | | |  | | | | |  | |
| 304 | | | Have you ever used any kind of substance before current pregnay? | 1.Yes 2.No | | | | | 2→309 | |
| 305 | | | Which kind? | 1.Alcohol 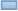yes 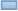 no  2.khat 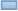yes 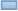 no  3.Any tobacco product 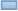yes 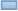 no  4.other/specify | | | | |  | |
| 306 | | | Have you ever used tobacco products? | 1.Yes 2.No | | | | |  | |
| 307 | | | Which type? | 1.Cigarrete 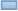yes 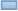 no  2.Chewing tobacco 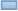yes 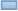 no  3.shisha 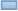yes 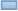 no  4.Other/specify | | | | |  | |
| 308 | | | Why did you use these substance/es? | 1.Socialization 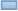 yes 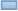 no  2.For excited way of life 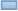yes 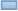 no 3.To manage life pressure 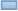yes 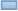 no 4.To get energy 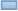 yes 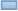 no  5.To boost appetite for food 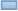 yes 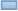 no  6.To obey tradition 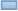 yes 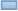 no 7.Others/specify | | | | |  | |
| **Part IV-Substance use during pregnancy** | | | |  | | | | |  | |
| 309 | | | Have you used any kind of substance during current pregnancy? | 1.Yes 2.No | | | | | 2→ 319 | |
| 310 | | | Which kind of substance? | 1.Alcohol | | | | | →311,312 | |
|  | | |  | 2.Khat | | | | | →313 | |
|  | | |  | 3.Tobacco product | | | | | →314,315 | |
|  | | |  | 4.others | | | | |  | |
| 311 | | | Type of alcohol use | 1.beer 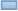 yes 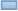 no  2.wine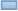 yes 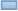 no  3.home made alcoholic drinks/specify | | | | |  | |
| 312 | | | How often do you drink alcohol? | 1. Monthly or less  2. 2–4 times a month  3. 2–3 times a week  4.4 or more times a week | | | | |  | |
| 313 | | | How often do you chew *khat?* | 1.every day  2.at least once a week,but not every day  3.less than once a week | | | | |  | |
| 314 | | | Type of tobacco product used | 1.Cigarrete 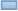yes 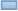 no  2.Chewing tobacco 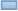yes 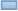 no  3.shisha 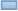yes 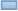 no  4.Other/specify | | | | |  | |
| 315 | | | How often do you use? | 1.every day  2.atleast once a week,but not every day  3.less than once aweek | | | | |  | |
| 316 | | | Why did you use these substance/es? | 1. Unaware of pregnancy 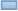 Yes 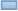 No  2. Unaware of its harm 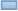 yes 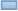 no  3. For socialization 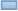 yes 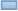 no  3. To cope up life pressure 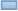yes 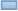 no  4.To obey tradition 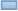yes 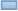 no  5.Others/specify | | | | |  | |
| 317 | | | What did you decide on substance use after recognition of pregnancy? | 1.To continue  2.To reduce dose  3.To reduce frequency  4.To stop | | | | |  | |
| 318 | | | Have you kept your decision? | 1.Yes 2.No | | | | |  | |
| **Part V-Partner substance use** | | | | | | | | | | |
| 319 | Is your partner use any type of substance? | | | 1.Yes 2.No | | | | | 2→321 | |
| 320 | Which type? | | | 1.Khat  2.Alcohol  3.Tobacco product  4.Other/specify | | | | |  | |
| **Part VI-Family Substance use** | | | |  | | | | |  | |
| 321 | Is there anyone using substance in the family? | | | 1.Yes 2.No | | | | |  | |
| 322 | Which type? | | | 1.Khat  2.Alcohol  3.Tobacco Product  4.Other/Specify | | | | |  | |
| **Patr VII-Intimate partner violence screening tool for pregnant women** | | | | | | | | | | |
|  | During the past 12 months has your husband/partner: | | | **Yes** | | | | | **No** | |
|  |  |  |  | Once | a few times | | Many times | |  | |
| 1 | Done things to scare or intimidate you on purpose? | | |  |  | |  | |  |  |
| 2 | Threatened to hurt you or someone you care about? | | |  |  | |  | |  | |
| 3 | Hit you, slapped you or thrown something at you that could hurt you? | | |  |  | |  | |  | |
| 4 | Forced you or pressured you to have sexual intercourse when you did not want to? | | |  |  | |  | |  | |
| During your current pregnancy, has your husband/partner: | | | | **Yes** | | | | | | **No** |
|  |  |  |  | Once | | A few times | | Many times | |  |
| 1 | | | Done things to scare or intimidate you on purpose? |  | |  | |  | |  |
| 2 | | | Threatened to hurt you or someone you care about? |  | |  | |  | |  |
| 3 | | | Hit you, slapped you or thrown something at you that could hurt you? |  | |  | |  | |  |
| 4 | | | Forced you or pressured you to have sexual intercourse when you did not want to? |  | |  | |  | |  |

**2. Amharic version questionnaire**

የአማርኛመጠይቅ

የቃለመጠይቅቀን (ቀን / ወር / ዓመት): _____________________________

የመረጃሰብሳቢስምእናፊርማ: ስም: ___________________ ፊርማ ___________________

ተቆጣጣሪስምእናፊርማ: ስም: ___________________ ፊርማ ___________________

የመጠይቁቁጥርኮድ: _____________________________

| ቁጥር | **ክፍል 1-** የማህበረሰብእናየስነሕዝብባህርያት | የምላሽአማራጮች | ይዝለሉ |
| --- | --- | --- | --- |
| 101 | እድሜ | _____ |  |
| 102 | ሃይማኖት | 1. ሙስሊም  2. ኦርቶዶክስ  3. ፕሮቴስታንት  4. ሌሎች ------------ |  |
| 103 | የመኖሪያቦታ | 1.ከተማ 2.ገጠር |  |
| 104 | ብሄር | --------------- |  |
| 105 | የጋብቻሁኔታ | 1. ያገባች  2. ያላገባች  3. ባሏየሞተባት  4. ከባሏየተፋታች  5. ያለጋብቻአብሮመኖር |  |
| 106 | የትምህርትደረጃ | 1. መጻፍእናማንበብየማይችል  2.መጻፍእናማንበብየሚችል  3.አንደኛደረጃ  4. ሁለተኛደረጃ  5.መሰናዶ  6.ኮሌጅእናከዚያበላይ |  |
| 107 | የሥራዎሁኔታ? | 1. ነጋዴ  2. የቤትእመቤት  3. ተማሪ  4. ሠራተኛ  5. ሌሎች ________ |  |
| 108 | የቤተሰብወርሃዊገቢ | ___________ |  |
| **ክፍል 2-** **ከእርግዝናእናወሊድጋርየተያያዙ** **ባሕርያት** | | |  |
| 201 | ምን ያህልጊዜ አርግዘሽታውቂያለሽ? | -------------- |  |
| 202 | ስንትልጆችአሉሽ? | -------------- |  |
| 203 | አሁን የስንትወርእርጉዝነሽ ? | ------------ - |  |
| 204 | እርግዝናሽ የታቀደ ነበር? | 1.አዎ 2.አይደለም |  |
| 205 | ምንያህልየእርግዝናክትትልአለሽ? | ________ |  |

| **ክፍል 3 - የእጽተጠቃሚነትተዛማጅባህሪያት** | | | | | |  |
| --- | --- | --- | --- | --- | --- | --- |
| **ግንዛቤንየተመለከቱጥያቄዎች** | | |  | | |  |
| 301 | በእርግዝናጊዜእጽመጠቀምየሚያስከትላቸውንጉዳቶችያውቃሉ? | | 1.አውቃለሁ  2. አላውቅም | | | 2→304 |
| 302 | ለጥያቄቁጥር “ 301 ” አውቃለሁካሉምንመጥፎተፅእኖዎችያውቃሉ? | | 1. ድንገተኛውርጃ   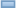 አዎ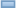አይ  2. ዝቅተኛክብደትያለውልጅመውለድ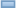አዎ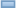አይ  3. አብሮየሚወለድ የልብችግር    አዎአይ  4.ያለግዜውየሚመጣምጥ    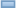 አዎ   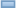 አይ  5. የአእምሮናየአካል   እድገት ችግር 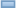 አዎ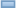አይ  6.    ሌላ / ይግለጹ | | |  |
| 303 | ይህንንመረጃከየትአገኙ? | | 1. ከጤናባለሙያዎች    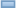 አዎ 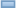 አይ  2.ከየመገናኛ ብዙሃን    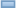 አዎ 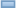አይ  3. ከመንደርሰው   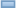 አዎ 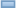 አይ  4 አላስታውስም | | |  |
| **ከእርግዝናበፊትየነበረየዕጽአጠቃቀምሁኔታ** | | |  | | |  |
| 304 | ከእርግዝናበፊትማንኛውንምአይነትእጽተጠቅመሽታውቂያለሽ? | | 1.አውቃለሁ  2.አላውቅም | | | 2→309 |
| 305 | የትኛውአይነት እጽ? | | 1. የአልኮል  መጠጥ     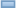  አዎ 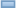 አይ  2.ጫት 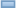አዎ 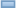 አይ  3.የትምባሆ ምርት    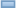አዎ 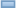 አይ   4. ሌላ / ይግለጹ | | |  |
| 306 | የትንባሆምርቶችንተጠቅመውያውቃሉ？ | | 1.አውቃለሁ  2.አላውቅም | | |  |
| 307 | ከተጠቀሙበየትኛውመልክ? | | 1.ሲጋራ     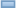 አዎ  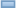አይ  2. የሚታኘክትንባሆ     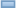 አዎ 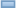 አይ  3.ሺሻ     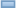 አዎ 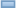 አይ  4. ሌላ / ይግለጹ | | |  |
| 308 | እነዚህንንጥረነገሮችየተጠቀማችሁበትምክንያቶች? | | 1.ለማህበራዊ  ኑሮ  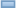 አዎ    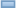 አይ  2. ደስተኛለመሆን 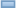አዎ 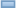 አይ  3. የኑሮንጫናለመቀነስ   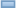 አዎ 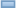 አይ  4. ኃይልለማግኘት    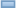አዎ 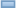አይ  5. የምግብፍላጎትንከፍለማድረግ  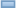አዎ 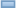 አይ  6. ባህልስለሆነ   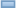 አዎ 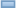 አይ  7 ሌሎች / ይግለጹ | | |  |
| **ክፍል 4-በእርግዝናጊዜየዕጽአጠቃቀምሁኔታ** | | | | | | |
| 309 | በአሁኑእርግዝናወቅትማንኛውንምአይነትዕጽተጠቅመውያውቃሉ? | | | 1.አውቃለሁ  2.አላውቅም | 2→319 | |
| 310 | የትኛውአይነትእጽ? | | | 1. የአልኮል  መጠጥ | →311, 312 | |
|  |  |  |  | 2.ጫት | →313 | |
|  |  |  |  | 3.የትምባሆ ምርት | →314, 315 | |
|  |  |  |  | 4. ሌላ / ይግለጹ |  | |
| 311 | የተጠቀሙትየመጠጥአይነት | | | 1.ቢራ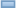 አዎ    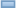 አይ  2.ወይን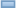 አዎ    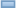 አይ  3.በቤትውስጥየሚዘጋጅ/ይጠቀስ |  | |
| 312 | በየስንትጊዜውይጠቀማሉ? | | | 1በወርአንዴወይምከዛባነሰ  2. በወርከ2–4ግዜ  3. በሳምንትከ2–3 ግዜ  4. በሳምንት4ግዜእናከዛበላይ |  | |
| 313 | በየስንትጊዜውይቅማሉ? | | | 1.በየቀኑ  2.ቢያንስበሳምንትአንዴ  3.በሳምንትከአንድጊዜባነሰ |  | |
| 314 | የተጠቀሙትየትንባሆአይነት | | | 1.ሲጋራ     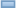  አዎ 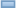 አይ   2. የሚታኘክትንባሆ   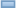  አዎ 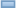 አይ  3.ሺሻ      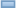  አዎ 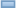 አይ  4. ሌላ / ይግለጹ |  | |
| 315 | በየስንትጊዜውይጠቀማሉ? | | | 1.በየቀኑ  2.ቢያንስበሳምንትአንዴ  3.በሳምንትከአንድጊዜባነሰ |  | |
| 316 | እነዚህንእጾችየተጠቀማችሁበትምክንያቶች? | | | 1. ነፍሰጡርመሆኔንባለማወቄ     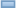  አዎ 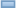 አይ  2.የሚያስከትለውንጉዳትባለማወቄ  አዎ  አይ  3. ለማህበራዊኑሮ         አዎ  አይ  4. የሕይወትንግፊትለመቋቋም   አዎ  አይ  5. ባህልስለሆነ     አዎ  አይ  6. ሌሎች / ይግለጹ |  | |
| 317 | እርግዝናከታወቀበኋላበእጽአጠቃቀምሁኔታዎላይምንወሰኑ? | | | 1. መቀጠል  2. መጠንለመቀነስ  3. ድግግሞሾቹንለመቀነስ  4. ለማቆም |  | |
| 318 | በውሳኔዎጸኑ？ | | | 1.አዎ  2.የለም |  | |
| **ክፍል-5የአጋርዎእጽየመጠቀምሁኔታ** | | | |  |  | |
| 319 | | ባለቤትዎማንኛውንምአይነትእጽይጠቀማል? | | 1.አዎ  2.የለም | 2→321 | |
| 320 | | የትኛውንአይነት  እጽ? | | 1. የአልኮል  መጠጥ       አዎ አይ  2.ጫት  አዎ  አይ  3.የትምባሆ ምርት     አዎ  አይ  4. ሌላ / ይግለጹ |  | |
| **ክፍል-6 የቤተሰብአባልእጽየመጠቀምሁኔታ** | | | |  |  | |
| 321 | | በቤተሰብውስጥእጽየሚጠቀም ሰውአለ? | | 1.አዎ  2.የለም |  | |
| 322 | | የትኛውንአይነት እጽ? | | 1. የአልኮል  መጠጥ       አዎ  አይ  2.ጫት አዎ  አይ  3.የትምባሆ ምርት    አዎ  አይ   4. ሌላ / ይግለጹ |  | |

| **ክፍል-**7 **ለነፍሰጡርሴቶችየተዘጋጀየአጋርጥቃትመለያቅጽ** | | | | | | | | |
| --- | --- | --- | --- | --- | --- | --- | --- | --- |
| ባለፉት12ወራትአጋርዎ፤ | | **አዎ** | | | | | **አይ** | |
|  |  | አንድግዜ | ጥቂትግዜ | | ብዙግዜ | |  | |
| 1 | ሆንብሎአስፈራርትዎትያውቃል? |  |  | |  | |  |  |
| 2 | እርስዎንወይምየሚወዱትንሰውጎድቶብዎያውቃል? |  |  | |  | |  | |
| 3 | መትቶዎት፣በጥፊመትቶዎትወይምሊጎዳዎትየሚችልእቃወርውሮብዎያውቃል? |  |  | |  | |  | |
| 4 | ሳይፈልጉየግብረስጋግንኙነትእንዲፈጽሙአስገድድዎትያውቃል? |  |  | |  | |  | |
| በዚህኛውእርግዝናወቅትባለቤትዎወይምአጋርዎ፤ | | **አዎ** | | | | | | **አይ** |
|  |  | አንድግዜ | | ጥቂትግዜ | | ብዙግዜ | |  |
| 1 | ሆንብሎአስፈራርትዎትያውቃል? |  | |  | |  | |  |
| 2 | እርስዎንወይምየሚወዱትንሰውጎድቶብዎያውቃል? |  | |  | |  | |  |
| 3 | መትቶዎት፣በጥፊመትቶዎትወይምሊጎዳዎትየሚችልእቃወርውሮብዎያውቃል? |  | |  | |  | |  |
| 4 | ሳይፈልጉየግብረስጋግንኙነትእንዲፈጽሙአስገድድዎትያውቃል? |  | |  | |  | |  |

**እናመሰግናለን !!!**

**3. Afan Oromo version data collection questionnaire**

Maqaa dhaabbata fayyaa ---------------------- Maqaa odeeffannoo funana-------------------------

Guyya odeeffannoon sun itti funaaname -----//--------//------- Kodii gaffii---------------

| **Kutaa:-I- Gaafiilee hawaasummaa fi haala jireenyaa ilaalatan** | | | |
| --- | --- | --- | --- |
| Lakk | Gaafiiwwan | Deebii | Irra dabrii |
| 101 | Umriiin kee meeqa? | Wagga---------- |  |
| 102 | Amantan kee maali | 1. Musliima 2. Ortodoksii 3. Prootestantii 4. Kan bira yoo tahee ibsii……. |  |
| 103 | Iddo jireenya kee essa | 1. Magaalaa 2. Baadiyyaa |  |

| 104 | Sabni kee malii | 1. Oromo 2. Amhara 3. Somalii 4. Kan biro yoo tahe ibsiii— |  |
| --- | --- | --- | --- |
| 105 | Haalli gaa’ela kee akkamii | 1. Kan heerumtee 2. Kan hin heerumin 3. Kan irraa du’ee 4. Kan hiiktee 5. Waliin jirachuu |  |
| 106 | Halii sadarka barumsa kee maal fakkata | 1. Bareessufi dubissu kan hin dandene 2. Bareessu fi dubbisu kan danda’an 3. Barumsa sad 1ffaa‘ 4. Barumsa Sadarka 2ffa 5. Barumsa qopha’ina 6. Barumsa kolleji + |  |
| 107 | Dalagan kee malli? | 1.Daldaltuu  2.Haadha manaa  3.barataa  4. Hojjataa mootumma/miti motuma  5.Kan biro……ibsi |  |
| 108 | Galin maati kee ji’aan meqaa | _______ |  |
| **Kutaa II: Gaaffiilee tajaajila sirna wal hormata haadhoolii ilaallatan** | | | |
| 201 | Hanga ammatii yeroo meqaa ulfoofte | Lakkoofsan--------- |  |
| 202 | Hanga ammatii yeroo meqaa desse | Lakkoofsan----- |  |
| 203 | Ulfaa ji’a meeqa gara qabda? | Lakkoofsan--------- |  |
| 204 | Ulfa kana karorfatani ulfooftani? | 1. Eyyeen 2. Lakki |  |
| 205 | Dahuu kee dura hordofii ulfaa meeqa qabda | ________ |  |
| **Kutaa III-Wanta sammuu nama hadochuu/haraara namati fidan kan ilaalatu** | | | |
|  | **Watota itti fayyadammu** |  |  |
| 301 | Yeroo ulfaa wantoota haraara/sammuu nama hadochan fayyadamuun midhaa namati fidu ni beektaa? | 1.Eyyeen  2.lakki | 2→304 |
| 302 | Deebin keesan eyyee yoo ta’e maal faadha? | 1.ulfii tasa namaraa bahuu  2.daa’ima ulfatinni isaa xiqqaa ta’e dhalachuu  3.dhibee onnee waliin dhalachuu  4.osoo yeroon hin gahin dhalachuu  5.Rakko sammuu wajjin dhalachuu  6.miidhaa bira |  |
| 303 | Odeefano kana essaa argatte? From where did you get this information? | 1.hojjataa fayyaa Eyyeen Lakki  2. sab,qunamtiEyyeen Lakki  3.ollaa irra Eyyeen Lakki  4.hin yaadadhu |  |
| **Oso hin ulfahin dura wanta samuu hadochu/harara namatti fidan fayyadamuu** | | | |
| 304 | Ulfaa’u kee dura wanta samu adochu/harara nama qabsisan fayadamaa turtee | 1.eyyen  2.lakki | 2→309 |
| 305 | Eyyen yoo tahee isa armann gadii kessa isaa kam? | 1.Alkooli dhuguu  2.Jimaa qama’uu  3.Shiishaa arsuu  4.Tamboo xuxuu  5.Kan biro……ibsi |  |
| 306 | Wanta Tamboo irraa omishaman fayyadamte ni beekta? (Have you ever used tobacco products?) | 1.Eyyen  2.Lakki |  |
| 307 | Isa kam fayadamtan? | 1.Sigaaraa  2.Tamboo  3.Shiishaa  4. Kan biro……ibsi |  |
| 308 | Maalif fayyadamtan? | 1.Walinjireenyaaf Eyyeen lakki  2. Ittin gammadudhaaf Eyyeen lakki  3.Dhiibbaa jireenya to’achuuf Eyyeen lakki  4.Humna argachuuf  Eyyeen Lakki  5.Fedhiin nyaataa akka dabaluuf Eyyeen lakki  6.Bartee/aadaa waan ta’eef Eyyeen lakki  7.kan biro…… ibsi |  |
| **Yeroo ulfaa wanta sammuu nama hadochuu/haraara nama qabsisu fayyadamuu** | | | |
| 309 | Ulfa kanarratti wanta harara nama qabsisan kamiyyuu fayyadamte beekta? | 1.Eyyeen  2.Lakki | 2→319 |
| 310 | Isa kami fayyadamte? | 1.Alkoolii/dhugaatii | →311,312 |
|  |  | 2.Jimaa | →313 |
|  |  | 3Wanta Tamboo irra oomishamu hundaa | →314,315 |
|  |  | 4.Kan biro_______ibsi |  |
| 311 | Gosaa dhugatii/alkoolii kami fayyadamte | 1.Biiraa eyyen lakki  2.waynii/ wine eyyen lakki  3. home made/ ibsii |  |
| 312 | Yeroo hagam hagamin alkoolii/dhugaatii dhuugda? | 1.ji’atti ykn ji’a gad  2.tara lama hanga afuritti torbaanitti  3.tara lama hanga sadi torbaanitti  4.tara afur ykn afur oli torbaanitti |  |
| 313 | Yeroo hagam hagamin jimaa qamaata? | 1. Guyyaa Guyyaadhaan  2. yoo tiqaate torbanittialitokoo garuu yeroo hunda miti  3. torbanitti altokkoo gadii |  |
| 314 | Gosa tamboo kami fayyadamtan? | 1.Sigaaraa Eyyeen Lakki  2.Tamboo alanfamEyyeen Lakki  3.shiishaa Eyyeen Lakki  4.Kan biro ibsi_____ |  |
| 315 | Yeroo hagam hagamin fayyadamta? | 1. Guyyaa Guyyaadhaan  2. yoo tiqaate torbanittialitokoo garuu yeroo hunda miti  3. torbanitti altokkoo gadii |  |
| 316 | Haraara kana maalif fayyadamte? | 1. Akkan ulfaa’e hin beekne Eyyeen Lakki  2. Midhaa isa hin beekuu Eyyeen Lakki  3. Walinjireenyaf Eyyeen Lakki  3. Dhiiba jireenya ittin irranfachuf/dabarsuuf  Eyyeen Lakki  4.Aadaa/bartee waan ta’ef  Eyyen Lakki  5.kan biro ibsi |  |
| 317 | Ulfa ta’uu kee erga bartee booda itti fayyadama haraaraa maali murteesite? | 1.itti fufu  2.hamma duraara baay’ina isaa hirisuu  3.dafani dafanii fudhachuu hirisuu  4.fudhachuu dhaabuu/dhiisuu |  |
| 318 | Murtii keessan fiitan ni baastani/hanga dhumatti itti fuftani jirtani? | 1.Eyyeen  2.Lakki |  |
| **Itti fayyadama haraara/wanta sammu nama hadochuu kana abbaa manaa** | | | |
| 319 | Abbaa mana kee haraara/wanta sammu nama hadochuu ni fayyadama? | 1.Eyyeen  2.Lakki | 2→321 |
| 320 | Gosa kami? | 1.Jimaa  2.alkoolii/dhugaatii  3.Wanta tamboo irraa oomishamu  4.kan biro ibsi |  |
| **Itti fayyadama haraara/wanta sammu nama hadochuu maatii** | | | |
| 321 | Maatii keessan keessa namni haraara/wanta sammu nama hadochuu fayadamu in jira? | 1.Eyyeen  2.Lakii |  |
| 322 | Gosa kami? | 1.Jimaa  2.alkoolii/Dhugaatii  3. wanta tamboo irraa oomishamu  4.kan biro ibsi |  |

| **Formii calalii hadhoole ulfa qabani dhibbaale abba manatin rawataman** | | | | | | |
| --- | --- | --- | --- | --- | --- | --- |
|  | Ji,oota 12 darban abba mana ykn hiriya qabdani | **Eyyeen** | | | | **Lakki** |
|  |  | Al tokko | yerroo muraasaf | | Yeroo hedduuf |  |
| 1 | Fayyida issatif sodaachisu ykn si dhiibu ture? |  |  | |  |  |
| 2 | Si miidhuf si sodaaachisa ture namni atti gargaartuf? |  |  | |  |  |
| 3 | Sirukkutu,si kabalu,ykn wanta biraa siratti darbuu kan si miidhu? |  |  | |  |  |
| 4 | Humnaan ykn dhiibbaan walqunamti saala akka gootu otto atti hin feene? |  |  | |  |  |
| Ulfa kee kan amman tanaa abba mana ykn hiriya qabdani | | **Eyyeen** | | | | **Lakki** |
|  |  | Al tokko | | yerroo muraasaf | Yeroo hedduuf |  |
| 1 | Fayyida issatif sodaachisu ykn si dhiibu ture? |  | |  |  |  |
| 2 | Si miidhuf si sodaaachisa ture namni atti gargaartuf? |  | |  |  |  |
| 3 | Sirukkutu,si kabalu,ykn wanta biraa siratti darbuu kan si miidhu? |  | |  |  |  |
| 4 | Humnaan ykn dhiibbaan walqunamti saala akka gootu otto atti hin feene? |  | |  |  |  |

**4. Af-somali version questionnaire**

Taariikhda waraysiga (taariik/bil/sanad):_____________________________

Magaca Keble: _______________________________

Magaca waraysi iyo saxiixaaga : Magaca: ___________________ saxiixa___________

Magaca kor- joogaha iyo saxiixaaga : Magaca: ___________________ saxiixa ___________Tirada codka su’aalaha ku: _____________________________

| **Lanbar** | **I-Badhitaan ka bulshada iyo arrimaha dadadweynaha** | **Jawaab** | | **Ugudub** |
| --- | --- | --- | --- | --- |
| 101 | Dh’dhadhu waa imissa? | ________ | |  |
| 102 | religion | 1. Muslim  2. Orthodox  3. Protestant  4. Others------------ | |  |
| 103 | Dagan | 1.Magalo  2.Miyi | |  |
| 104 | Gomiyaad | _______ | |  |
| 105 | Xaalada guuree wakhtigan xadirka ah | 1.Gursaday  2.Magursaan  3.Ninkeedi ka dhintay  4.Carmac  5. Wadaa nolasho | |  |
| 106 | Herka waxbarasho | 1.Ma akrin karaa oo qori  2.Akhrin karaa oo qori  3.Dugsi hoose  4.Dugsisaare  5.Dhugsi u diyaarin  6.College oo kor ku xusan | |  |
| 107 | Shaqada | 1.Ganacasad  2.Gurii jog  3.Arday  4.Shaqale  5.Waxkale | |  |
| 108 | Lacagta soogasha xafada bishii? |  | |  |
| **Su’alaha la xidhidha xiliga uurka iyo dhalmada** | | | | |
| 201 | Intee jeer baad uraysatay? | ___________ | |  |
| 202 | Imisa carur badledahay? | ___________ | |  |
| 203 | Intee billod ayaadtahay? | ___________ | |  |
| 204 | Madkutalagashay uurkan? | 1.haa  2.maya | |  |
| 205 | Imasa jeer ayadtagtay adeega uurlaydha? | ________ | |  |
| **Dabeecadaha la xidhidha buwadahamukhaadaradka** | | | | |
| **Sucalaha la xidheeda dhiyagarowgha** | | | | |
| 301 | Mukhaadarahakataarta ku ah xiliga uurka madgaranaysa? | 1.haa  2.maya | | 2→304 |
|  |  |  | |  |
| 302 | Hadad garanayso, maxa kamid ah? | 1.dhicis haa maya  2.culayska illmo yar haa maya  3.wadha xanunka iyu kudhashay  haa maya  4. Aan shinkisii gadhin haamaya  5.hannunadha nervaha kufafah  haa maya  6. waxkale/kalasaar | |  |
| 303 | Hoogta hageed kaheshay? | 1.xirfadle cafimad haa maya  2.waarbahinta haa maya  3.hafadhaha haa maya  4.maxasusto | |  |
| MukhaadaradkaUurka hortisa lacisticimalo | | | | |
| 304 | Mukhaadaraha waliga urka kehore Macistimashay? | 1.haa  2.maya | | 2→309 |
| 305 | Noceed esticimashay | 1.khamro haa maya  2.Jadh haa maya  3.tobaco haa maya  4.waxkale/kalasaar | |  |
| 306 | Waliga tobacco madcisticmashay? | 1.haa  2.maya | | 2→308 |
| 307 | Noceed esticimashay? | 1.sigaar haa maya  2.tubaakadalacalasha haa maya  3.xashiishad haa maya  4.waxkale | |  |
| 308 | Maxaad uu esticimashamukhaadaradka? | 1.bulshawinimo haa maya  2.faraxad haa maya  3.in aan kujahoxaladaha adeg  haa maya  4.in aan kahelo awood haamaya  5.in uu iifuro cuntada haa maya  6. in aan raaco dhaqankayga  haa maya  7. waxkale/kalasaar | |  |
| Mukhaadaraha la cisticmaalo xiliga uurka | | | | |
| 309 | Ma esticmashay wax mandoriya intaad uurkanlahayd? | 1.haa 2.maya | 2→319 | |
| 310 | Maandooriye nocee ah? | 1.khamro | →311, 312 | |
|  |  | 2.Jadh | →313 | |
|  |  | 3.tobaco | →314, 315 | |
|  |  | 4.waxkale/kalasaar |  | |
| 311 | Nooca khamriga loo esticmaalo | 1.beer haa maya  2.wine haa maya  3.degaanka diyaariyey/kalasaar |  | |
| 312 | Imisa jeer ayaad cabtaa khamriga? | 1. bil kasta ama ka yar  2. 2–4 jeer bishii  3. 2–3 jeer toddobaadka  4.4 jeer am aka badan toddobaadkii |  | |
| 313 | Imisa jeer aad cunaan jaadka? | 1.Maalin walba  2. Ugu yaraan hal mar todobaadkii, laakiin aan maalin kasta.  3.In ka yar hal mar ka badan toddobaadkii |  | |
| 314 | Nooca waxyaabaha tubaaakada loo isticmaalo | 1.Sigaar  2.Tubaakada lacalashado  3.Xashiishad  4. waxkale/kalasaar |  | |
| 315 | Imisa jeer aad isticmaasho? | 1.Maalin walba  2. Ugu yaraan hal mar todobaadkii, laakiin aan maalin kasta.  3.In ka yar hal mar ka badan toddobaadkii |  | |
| 316 | Maxaad uu esticimasha mukhaadaradkan? | 1.Maan ogayn uurka  2.Man garanayn dhibatadeeda  3. Bulshawinimo  4.In aan kujaho xaladaha adeg  5. In aan raaco dhaqankayga  6. Waxkale/kalasaar |  | |
| 317 | Maxad samaysay markaad ogaatay in aad uur leeday | 1.waan siiwaday  2.wan yaraystay  3.xiliyadi ann isticmali jiray ayaan yareeyay  4.waan joojiyay |  | |
| 318 | Maku adkaysatay go’aan kaagii | 1.haa 2.maya |  | |
| **Isticimalka mandoriyaha ee ninkaga** | | | | |
| 319 | Ninkaga muu isticimaala mukhaadaradkan? | 1.haa  2.maya | 2→321 | |
| 320 | Noocee? | 1.khamro  2.Jadh  3.tobaco  4.waxkale/kalasaar |  | |
| **Isticmaalka qoyska ee mukhaadaradka** | | | | |
| 321 | Majirtaa cid isticmasha mandooriyaha  Qoyskaga? | 1.haa  2.maya |  | |
| 322 | Noocee? | 1.khamro  2.Jadh  3.tobaco  4.waxkale/kalasaar |  | |

| **Foomka xog uruurinta Caadidska odayga ee urlaydaa** | | | | | | |
| --- | --- | --- | --- | --- | --- | --- |
|  | 12 kii bilood ee lasodhaafay miyyu odaygaaga amma saaxibka kula oogay | **Haa** | | | | **Maya** |
|  |  | Hal mar | wax yar | | Inbadan |  |
| 1 | Faaidadisa dartis miyu ku cabsiyay ama ku cadaadiyay? |  |  | |  |  |
| 2 | Qof aad daneeynayso miyu ku cadaaadiyay? |  |  | |  |  |
| 3 | Ma ku garaacay, ma kudharbaaxay amma wax ku dhiba makuguso turay? |  |  | |  |  |
| 4 | Adon ubahnayn cadaadis amma xoog inu kula galmoodo masameeyay? |  |  | |  |  |
| Uurkaagan hada miyu odaygaga amma saaxibka kula joogay: | | **Haa** | | | | **Maya** |
|  |  | Hal mar | wax yar | Inbadan | |  |
| 1 | Faaidadisa dartis miyu ku cabsiyay ama ku cadaadiyay? |  |  |  | |  |
| 2 | Qof aad daneeynayso miyu ku cadaaadiyay? |  |  |  | |  |
| 3 | Ma ku garaacay, ma kudharbaaxay amma wax ku dhiba makuguso turay? |  |  |  | |  |
| 4 | Adon ubahnayn cadaadis amma xoog inu kula galmoodo masameeyay? |  |  |  | |  |

AAD AYAD UMAHADSANTAHAY!!
